# Supplementary material for: Mitogenomic phylogeny of Tetrigoidea (Insecta, Orthoptera), with a focus on the genus Zhengitettix
Source: PeerJ. 2025 Jun 9;13:e19521. doi: 10.7717/peerj.19521 (PMC12161130; doi:10.7717/peerj.19521)
Supplement: Supplemental Information 1 [file peerj-13-19521-s001.docx]

**Figure S1 The nucleotide composition in two sampled Tetrigoidea species.** Note: a: nucleotide content in *Zhengitettix curvispinus*; b: base skew in *Zhengitettix curvispinus*; c: nucleotide content in *Scelimena melli*; d: base skew in *Scelimena melli*; PCG-1st: the first position of codons in PCGs; PCG-2nd: the second position of codons in PCGs; PCG-3rd: the third position of codons in PCGs.

*
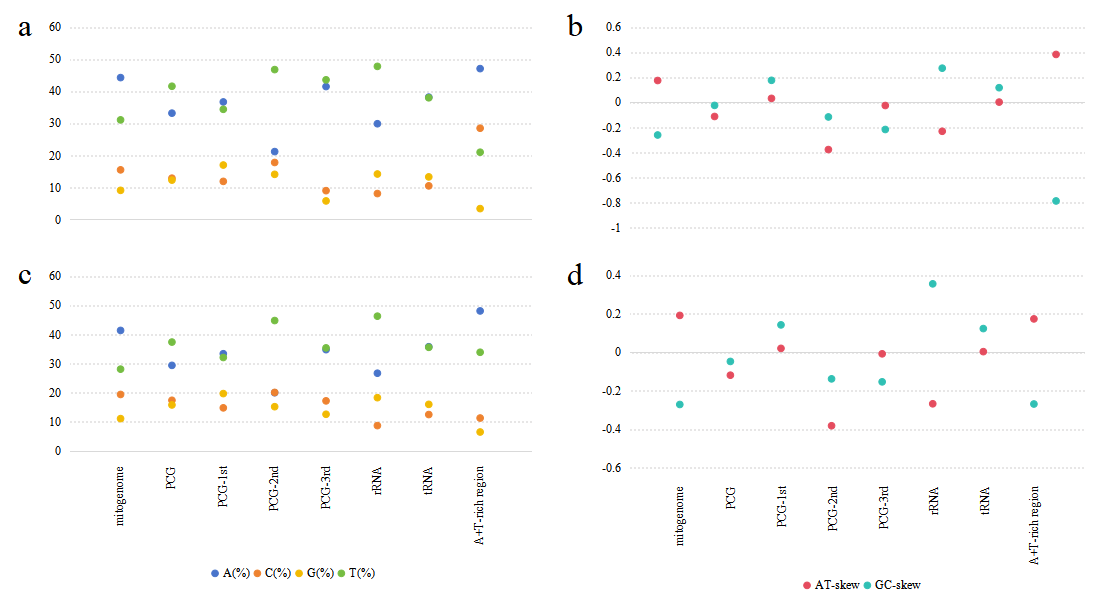
*

**Figure S2 The types of initiation and termination codons in 50 Tetrigoidea mitogenomes.** Note: a: initiation codon, b: termination codon.


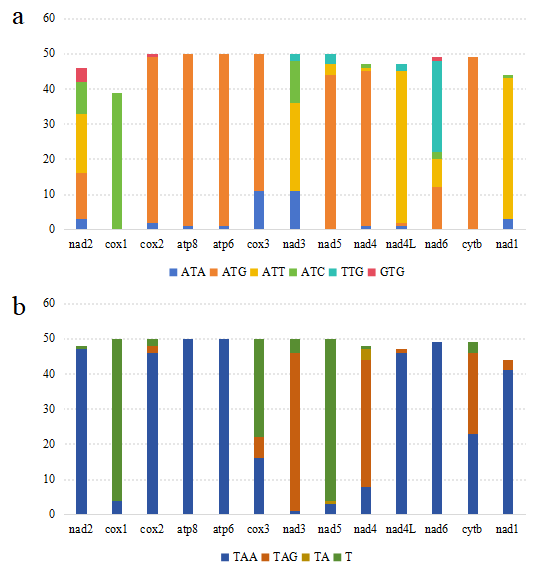


**Figure S3 The secondary structure of ser(agn) in *Zhengitettix curvispinus*.**


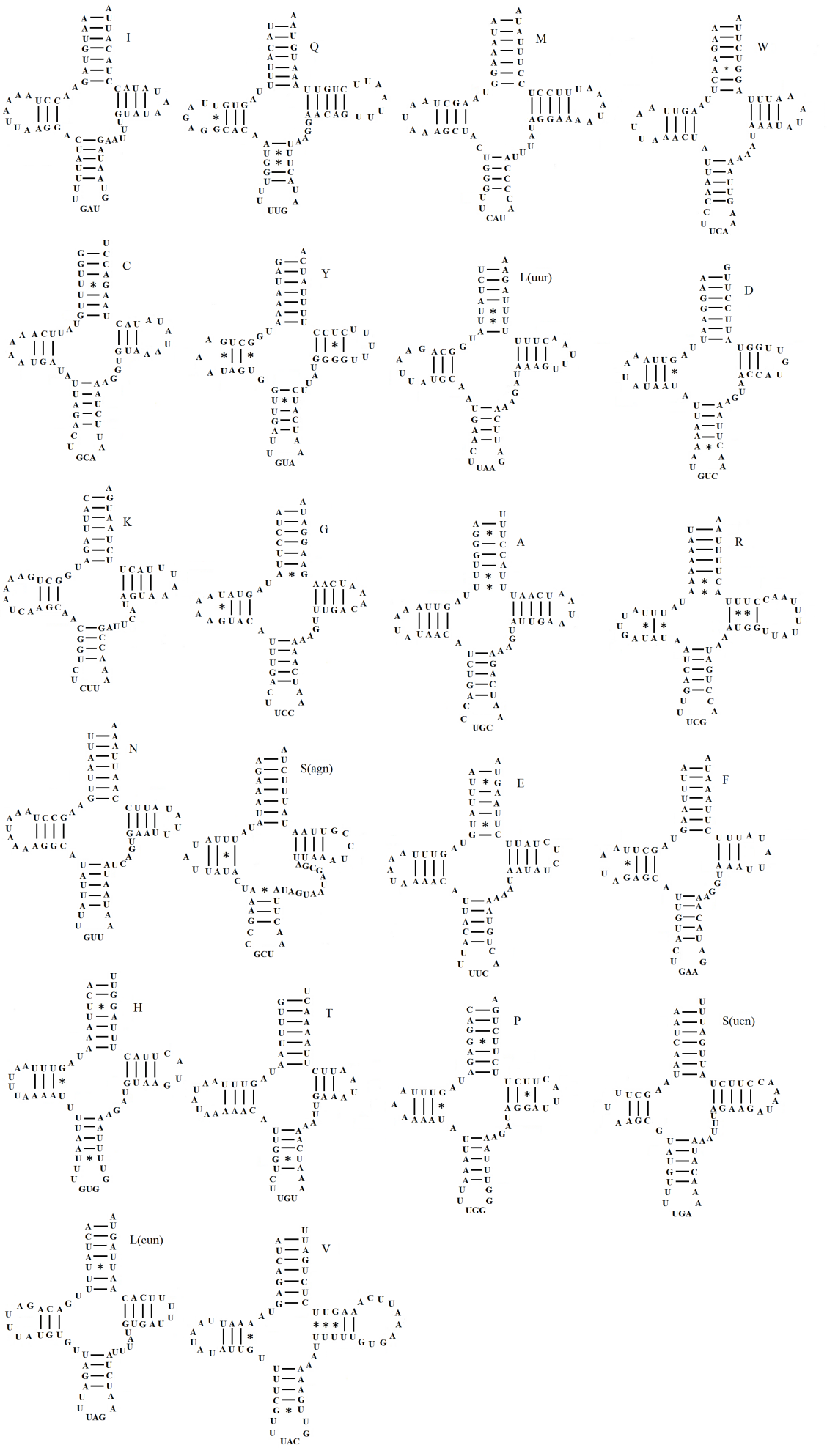


**Figure S4 The secondary structure of ser(agn) in four other sampled Tetrigoidea species.** Note: a: *Scelimena melli*, b: *Zhengitettix hainanensis*, d: *Eucriotettix oculatus*, d: *Thoradonta yunnana*.


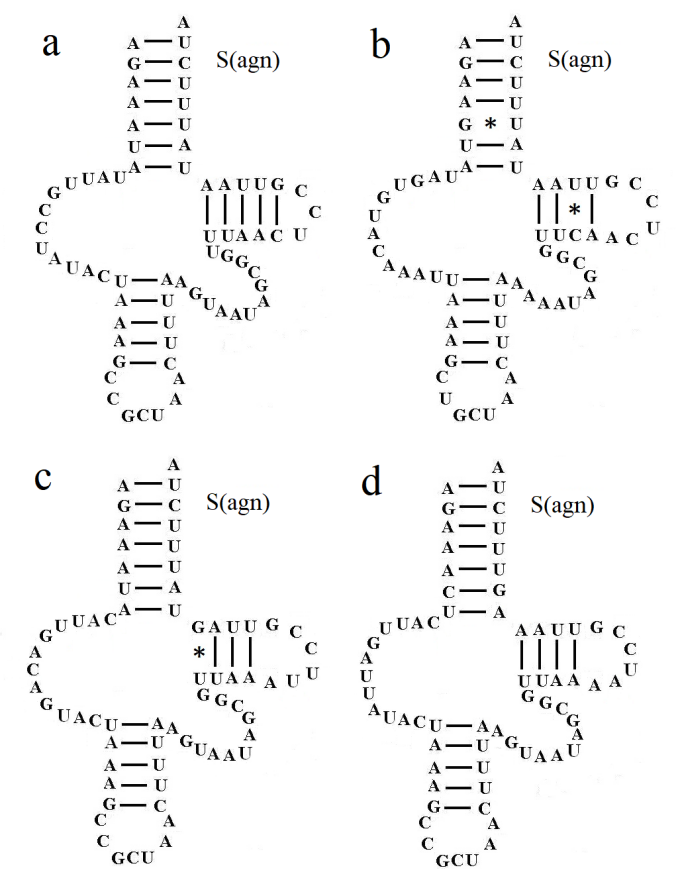


**Figure S5 The saturation test of three datasets.** Note: a: PCG, b: PCG_rRNA, c: PCG_rRNA_tRNA.

**
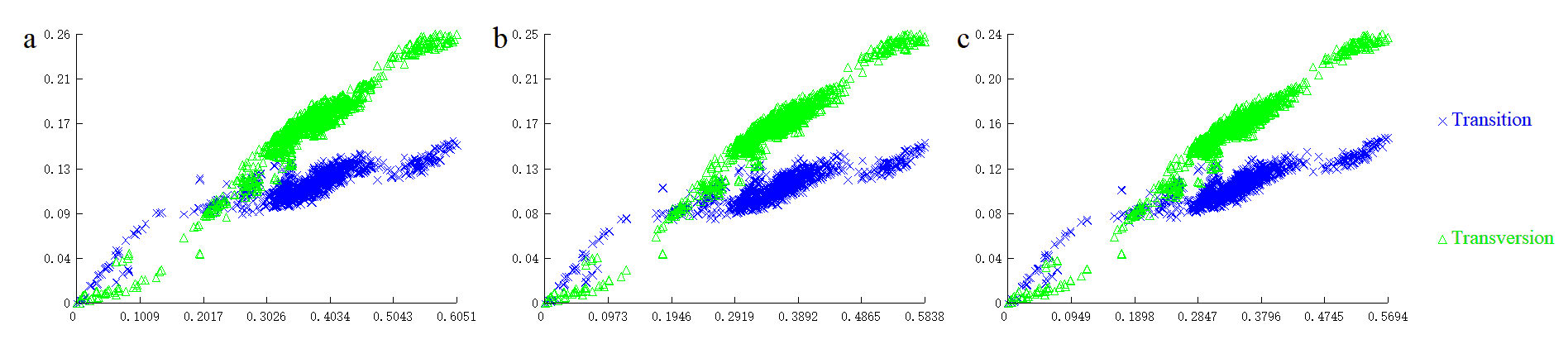
**

**Figure S6 The phylogenetic trees of three datasets based on ML and BI methods, corresponding to Figure 3.** Note: a: PCG ML tree, b: PCG BI tree, c: PCG_rRNA ML tree, d: PCG_rRNA BI tree, e: PCG_rRNA_tRNA ML tree, f: PCG_rRNA_tRNA BI tree.


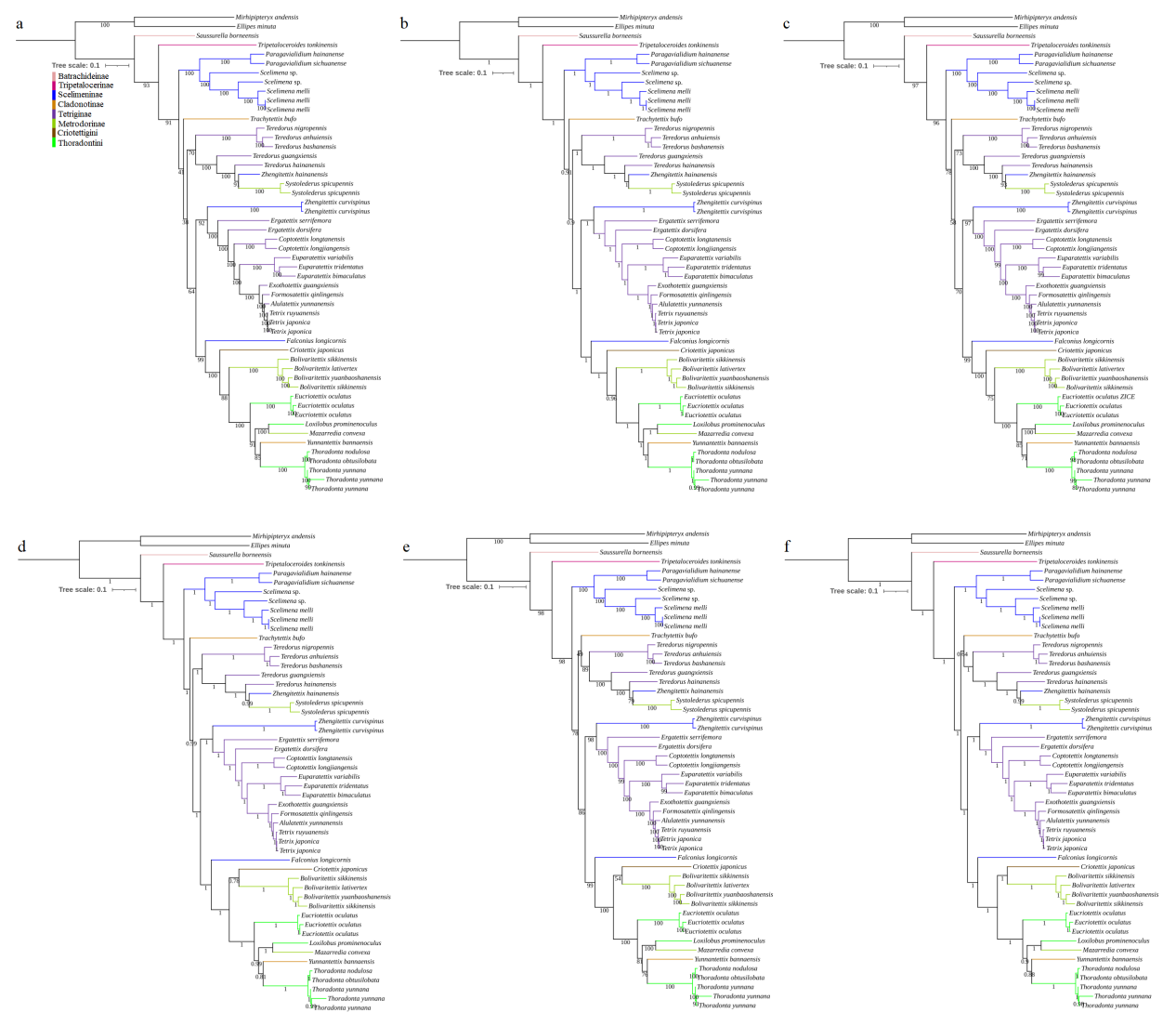


**Figure S7 The p-distance result between *Zhengitettix hainanensis* and other closely related *Zhengitettix*, *Teredorus* and *Systolederus* species based on the cox1 gene.**


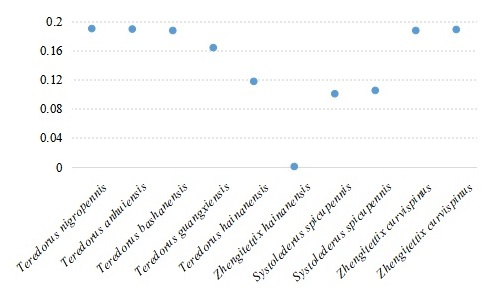


**Figure S8 The phylogenetic trees associated with the genus** *Zhengitettix*. Note: a: PCG ML tree, b: PCG BI tree.

**
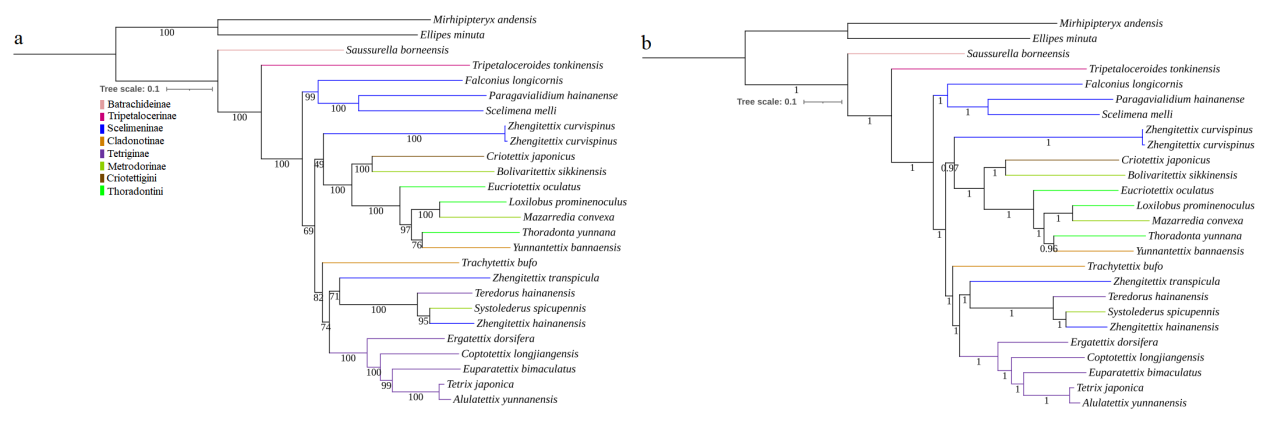
**

**Table S1 The mitogenomic sequence data used for reconstructing phylogenetic trees.** Note: the mitogenome data in this study labeled in red font color; #: the mitogenome sequences used for Tetrigoidea phylogeny; *: the mitogenome sequences used for phylogenetic analyses of the genus *Zhengitettix*.

| Classification | Species | Accession number | Analysis |
| --- | --- | --- | --- |
| Batrachideinae | *Saussurella borneensis* | MZ169555 | # * |
| Cladonotinae | *Trachytettix bufo* | JX913766 | # * |
| Cladonotinae | *Yunnantettix bannaensis* | MN083181 | # * |
| Criotettigini | *Criotettix japonicus* | MT162542 | # * |
| Metrodorinae | *Bolivaritettix lativertex* | MN083173 | # |
| Metrodorinae | *Bolivaritettix sikkinensis* | KY123120 | # * |
| Metrodorinae | *Bolivaritettix sikkinensis* | MN083174 | # |
| Metrodorinae | *Bolivaritettix yuanbaoshanensis* | KY123121 | # |
| Metrodorinae | *Mazarredia convexa* | MN938924 | # * |
| Metrodorinae | *Systolederus spicupennis* | MN083179 | # * |
| Metrodorinae | *Systolederus spicupennis* | MH791445 | # |
| Scelimeninae | *Falconius longicornis* | MT162543 | # * |
| Scelimeninae | *Paragavialidium hainanense* | NC_071831 | # * |
| Scelimeninae | *Paragavialidium sichuanense* | MT162549 | # |
| Scelimeninae | *Scelimena melli* | MT162548 | # |
| Scelimeninae | *Scelimena melli* | MW722938 | # |
| Scelimeninae | *Scelimena melli* | PQ783660 | # * |
| Scelimeninae | *Scelimena sp.* | OR333957 | # |
| Scelimeninae | *Scelimena sp.* | OP057410 | # |
| Scelimeninae | *Zhengitettix curvispinus* | MT162544 | # * |
| Scelimeninae | *Zhengitettix curvispinus* | PQ783659 | # * |
| Scelimeninae | *Zhengitettix hainanensis* | PQ783661 | # * |
| Scelimeninae | *Zhengitettix transpicula* | PQ869509 | * |
| Tetriginae | *Alulatettix yunnanensis* | NC_018542 | # * |
| Tetriginae | *Coptotettix longjiangensis* | KY798413 | # * |
| Tetriginae | *Coptotettix longtanensis* | OK540319 | # |
| Tetriginae | *Ergatettix dorsifera* | NC_046540 | # * |
| Tetriginae | *Ergatettix serrifemora* | MN938923 | # |
| Tetriginae | *Euparatettix bimaculatus* | NC_046541 | # * |
| Tetriginae | *Euparatettix tridentatus* | NC_082933 | # |
| Tetriginae | *Euparatettix variabilis* | NC_046542 | # |
| Tetriginae | *Exothotettix guangxiensis* | NC_082934 | # |
| Tetriginae | *Formosatettix qinlingensis* | KY798412 | # |
| Tetriginae | *Teredorus anhuiensis* | NC_071822 | # |
| Tetriginae | *Teredorus bashanensis* | NC_063118 | # |
| Tetriginae | *Teredorus guangxiensis* | NC_082935 | # |
| Tetriginae | *Teredorus hainanensis* | NC_063117 | # * |
| Tetriginae | *Teredorus nigropennis* | MN938922 | # |
| Tetriginae | *Tetrix japonica* | NC_018543 | # * |
| Tetriginae | *Tetrix japonica* | EU623447 | # |
| Tetriginae | *Tetrix ruyuanensis* | NC_046412 | # |
| Thoradontini | *Eucriotettix oculatus* | MT162546 | # |
| Thoradontini | *Eucriotettix oculatus* | MN083176 | # |
| Thoradontini | *Eucriotettix oculatus* | PQ783662 | # * |
| Thoradontini | *Loxilobus prominenoculus* | MT162545 | # * |
| Thoradontini | *Thoradonta nodulosa* | MT162547 | # |
| Thoradontini | *Thoradonta obtusilobata* | KY798414 | # |
| Thoradontini | *Thoradonta yunnana* | NC_071832 | # |
| Thoradontini | *Thoradonta yunnana* | OP805341 | # |
| Thoradontini | *Thoradonta yunnana* | PQ783663 | # * |
| Tripetalocerinae | *Tripetaloceroides tonkinensis* | MW770353 | # * |
| Outgroup(Tridactyloidea) | *Ellipes minuta* | NC_014488 | # * |
| Outgroup(Tridactyloidea) | *Mirhipipteryx andensis* | NC_028065 | # * |

**Table S2 The mitogenome sequencing information.**

| Species | Reads number of raw data | Reads number after trimming | Reads number used for mitogenome assembly |
| --- | --- | --- | --- |
| *Zhengitettix curvispinus* | 18,875,283 | 18,438,039 | 18,438,039 |
| *Zhengitettix hainanensis* | 318,704,454 | 310,007,971 | 24,800,638 |
| *Scelimena melli* | 284,517,431 | 277,192,283 | 23,838,537 |
| *Eucriotettix oculatus* | 329,346,349 | 319,263,530 | 23,944,765 |
| *Thoradonta yunnana* | 230,578,190 | 216,059,448 | 22,902,301 |

**Table S3 The tandem repeats in species of the genus *Zhengitettix* and *Scelimena*.**

| Species | Tandem repeat | Indices | Copy number | Consensus size | Consensus pattern |
| --- | --- | --- | --- | --- | --- |
| *Zhengitettix curvispinus* (PQ783659) | TR1 | [109-1075](https://tandem.bu.edu/trf/output/tmpt5cw6_yk.2.7.7.80.10.50.500.1.txt.html#109--1075,59,16.4,59,1) | 16.4 | 59 | ATAAATTTTCAACCCCAAATCAAAAAATCCTAACCCTGTCCTCCACAAAACAAGATCAA |
| *Zhengitettix curvispinus* (MT162544) | TR1 | [136-666](https://tandem.bu.edu/trf/output/tmpqc2yi0rx.2.7.7.80.10.50.500.1.txt.html#136--666,58,9.2,58,1) | 9.2 | 58 | AAATTCTGCTCTTATTCTCCACAAAACAAGATCAAATAAATTATTAACCCAAATCAAC |
|  | TR2 | [147-296](https://tandem.bu.edu/trf/output/tmpqc2yi0rx.2.7.7.80.10.50.500.1.txt.html#147--296,29,5.2,29,2) | 5.2 | 29 | TTATTCTCCACAAAACAAGATCAAATAAA |
| *Zhengitettix transpicula* | TR1 | 31-73 | 2.2 | 20 | AAAATCATAAAATAAAAAGA |
| *Scelimena melli* (PQ783660) | TR1 | [898-1498](https://tandem.bu.edu/trf/output/tmpuglsyywo.2.7.7.80.10.50.500.1.txt.html#898--1498,213,2.8,215,1) | 2.8 | 215 | TATTAAATAAATATTTAAATATAAAATAAATTCTAATCTATATAATACAATAAATGATTATACATATAGAATTATCTTATATATTAGAATATAATTATATATATGTATAATAAGTGTTTATTTTAAATAATATTACATAACTTATAATAAGTACTTATTATATAATAGTTCTCTTAATCTCCTACAATAATGCTATACTATATATAGATTAGTTA |
| *Scelimena* sp. (OR333957) | TR1 | [905-1368](https://tandem.bu.edu/trf/output/tmpcz7rstuo.2.7.7.80.10.50.500.1.txt.html#905--1368,212,2.2,217,1) | 2.2 | 217 | ATTATATTAAATAAATATTTAAATATAAAATAAATTCCAATCCTATATAATGCAATAAATAATTATATTTATAAAAAATATATTATACAATAATATATGAATGTATATACATTACAATAAGTATTTATTTTAAATAATACTAAATAACTACCAATAAGCACTTATTATAAAATATGCTCTTAATCTCCTAAAATAATGCTATACTATATATAGATAA |
| *Scelimena* sp. (OP057410) | TR1 | [53-95](https://tandem.bu.edu/trf/output/tmpod0k12h_.2.7.7.80.10.50.500.1.txt.html#53--95,12,3.3,13,2) | 3.3 | 13 | AAAATAAAAAATA |
|  | TR2 | [53-89](https://tandem.bu.edu/trf/output/tmpod0k12h_.2.7.7.80.10.50.500.1.txt.html#53--89,7,5.4,7,3) | 5.4 | 7 | AAAATAA |
|  | TR3 | [878-924](https://tandem.bu.edu/trf/output/tmpod0k12h_.2.7.7.80.10.50.500.1.txt.html#878--924,23,2.0,23,4) | 2.0 | 23 | AATAAAATATATAATTACAATTA |
|  | TR4 | [963-1863](https://tandem.bu.edu/trf/output/tmpod0k12h_.2.7.7.80.10.50.500.1.txt.html#963--1863,213,4.2,213,5) | 4.2 | 213 | TTATTTTAAATAGATTGTATACATTAAATAAGTACTTATTATAAAATATTCTCTTAATCTCTCAAAATAATGCTATTCTATGTATAGATTAAATATATTAAATAAATATTTAAATATAAAATAAATCCAAGTCGCTATAATATAATAGATAATTAGATATAAATAATTATCTTATATATTTATATATTAATGTATATACCTAACAATAAGTAC |
|  | TR5 | [1476-1514](https://tandem.bu.edu/trf/output/tmpod0k12h_.2.7.7.80.10.50.500.1.txt.html#1476--1514,21,1.9,20,7) | 2.0 | 20 | ATTAAATATATAAATAAATA |
|  | TR6 | [2276-2316](https://tandem.bu.edu/trf/output/tmpod0k12h_.2.7.7.80.10.50.500.1.txt.html#2276--2316,17,2.5,17,9) | 2.5 | 17 | TAACTAAACTTAACATT |
|  | TR7 | [2529-2569](https://tandem.bu.edu/trf/output/tmpod0k12h_.2.7.7.80.10.50.500.1.txt.html#2529--2569,17,2.5,17,11) | 2.5 | 17 | TAACTAAACTTAACATT |
|  | TR8 | [2100-2936](https://tandem.bu.edu/trf/output/tmpod0k12h_.2.7.7.80.10.50.500.1.txt.html#2100--2936,253,3.3,253,12) | 3.3 | 253 | AAAAATACTACCTCCAGTTGAACTATAAGAAAGTACAAAACCCTAAATAATGACTAGATAAACCTAATTATCTTAATTAAATCTTAACTTTAATTTCAAACAAATACAGCCAATAAATAAATCCTACAGTAAAACTCTATGCCCCCTAGAGCAATACTAGTATATACCCTAACAACTAACAAACTTAACATTTAACTAAAGTTAACCTTTAACTAAAATAAGTTCCCGCTTTTGTATCCACAAAAAAAGCGGG |
|  | TR9 | [2782-2822](https://tandem.bu.edu/trf/output/tmpod0k12h_.2.7.7.80.10.50.500.1.txt.html#2782--2822,17,2.5,17,14) | 2.5 | 17 | TAACTAAACTTAACATT |
